# Online learning for WHO priority diseases with pandemic potential: evidence from existing courses and preparing for Disease X

**DOI:** 10.1186/s13690-023-01080-9

**Published:** 2023-04-21

**Authors:** Heini Utunen, Anna Tokar, Mafalda Dancante, Corentin Piroux

**Affiliations:** Learning and Capacity Development Unit, Health Emergencies Programme, World Health Organization, Avenue Appia 20, 1202 Geneva, Switzerland

**Keywords:** Online-learning, Pandemic, Infectious disease, Health emergencies, Language

## Abstract

**Background:**

OpenWHO is the open-access learning platform of the World Health Organization (WHO) that provides online learning for health emergencies with essential health knowledge for emergencies. There is emphasis for courses on severe emerging diseases with epidemic and pandemic potential to help frontline health workers prevent, control and respond to infectious diseases. This research addresses the question of how the existing OpenWHO online courses on infectious disease were used in the countries of disease occurrence and how to prepare for disease X, a novel or unknown pathogen with pandemic potential.

**Methods:**

OpenWHO collects self-declared demographic data from learners among which there is data on geographical location of learners. Data in infectious disease courses use on OpenWHO was collected and examined and additionally information languages used in the outbreak locations was collected.

**Results:**

For most diseases in focus the online learning materials were used in countries with burden of disease. This suggests the learning material production needs to be targeted for outbreak and epidemic events.

**Conclusions:**

Findings inform the use of learning materials in disease outbreaks. Further, this use case data confirms learning providers need to add offerings in languages spoken in outbreak impacted areas.

## Background

OpenWHO is an open-access, low-bandwidth online learning platform that has massively expanded its reach during the COVID-19 pandemic [53, 59]. The mission of the platform is to provide online life-saving knowledge based on the latest WHO technical guidance to frontline responders, policymakers, and the public. The platform hosts courses on 33 different infectious diseases in 65 languages aiming to produce critical learning materials for any health emergency events and outbreak to which WHO responds. To serve the need for online learning in health emergencies the OpenWHO platform was launched in 2017 following the Ebola West Africa outbreak lessons learnt, in which a low-bandwidth adjusted learning platform became a bespoke need.

OpenWHO courses focus on diseases listed in WHO’s Research and Development (R&D) Blueprint as priority diseases. This WHO list distinguishes which diseases pose the greatest public health risk due to their epidemic potential and/or whether there are no or insufficient countermeasures. The list of currently severe emerging diseases with epidemic and pandemic potential include: COVID-19, Crimean Congo hemorrhagic fever, Ebola virus disease, and Marburg, Lassa fever, MERS and SARS coronavirus diseases, Zika, Nipah, Rift Valley fever and Disease X. Per WHO, this is not an exhaustive list, nor does it indicate the most likely causes of the next pandemic. The list is reviewed annually or when new diseases emerge.

**Fig. 1:**
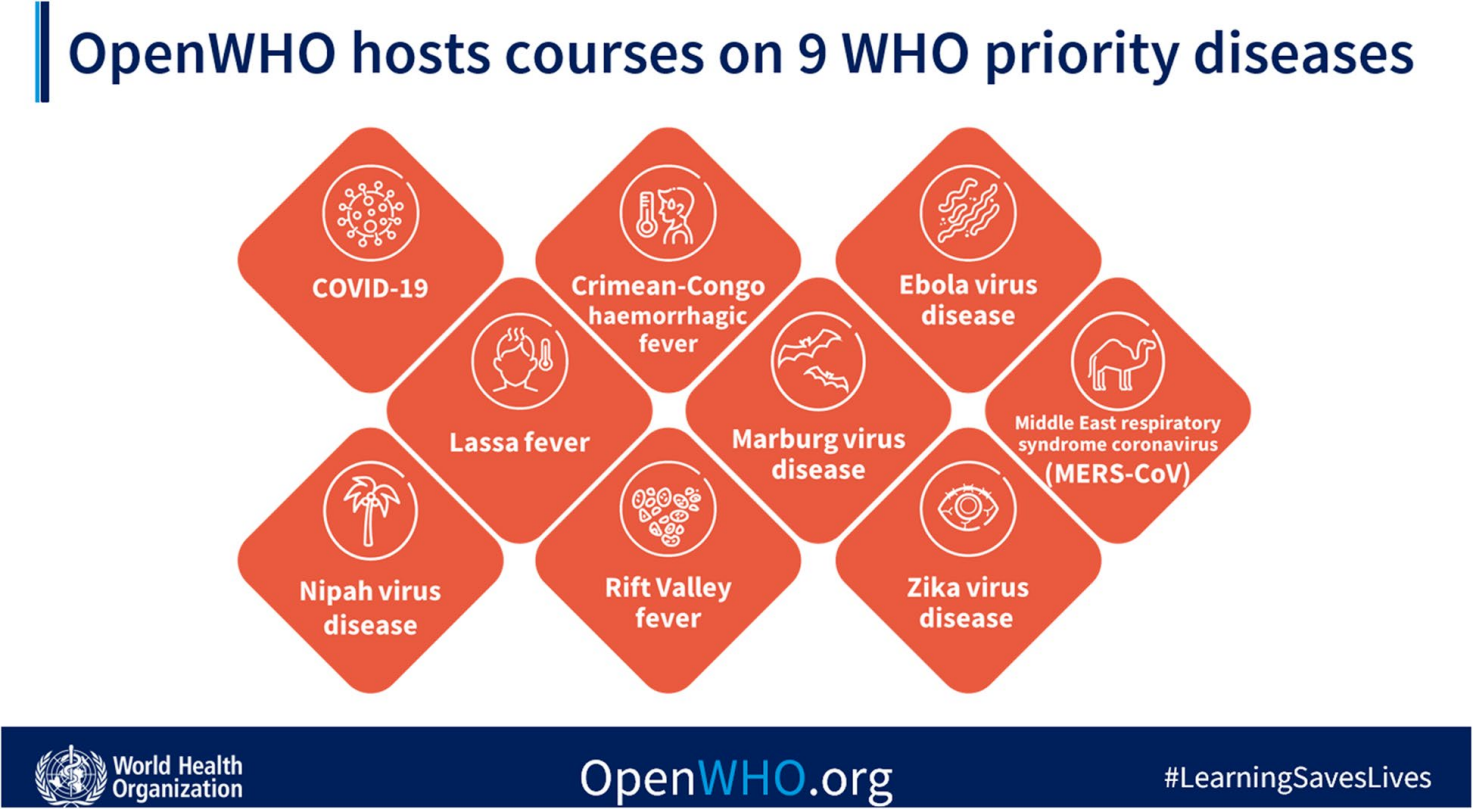
OpenWHO courses on 9 WHO priority diseases for pandemic potential

According to WHO, Disease X is *a serious international epidemic caused by a pathogen currently unknown to cause human disease* [61]. COVID-19 was a Disease X. The list is being updated frequently. OpenWHO hosts learning for all diseases of the 2022 list of severe emerging diseases with epidemic and pandemic potential. There are in total 46 different courses for the COVID-19 disease, 5 for Ebola, and at least one course for each of the other diseases of the list (Fig. 1).

**Fig. 2:**
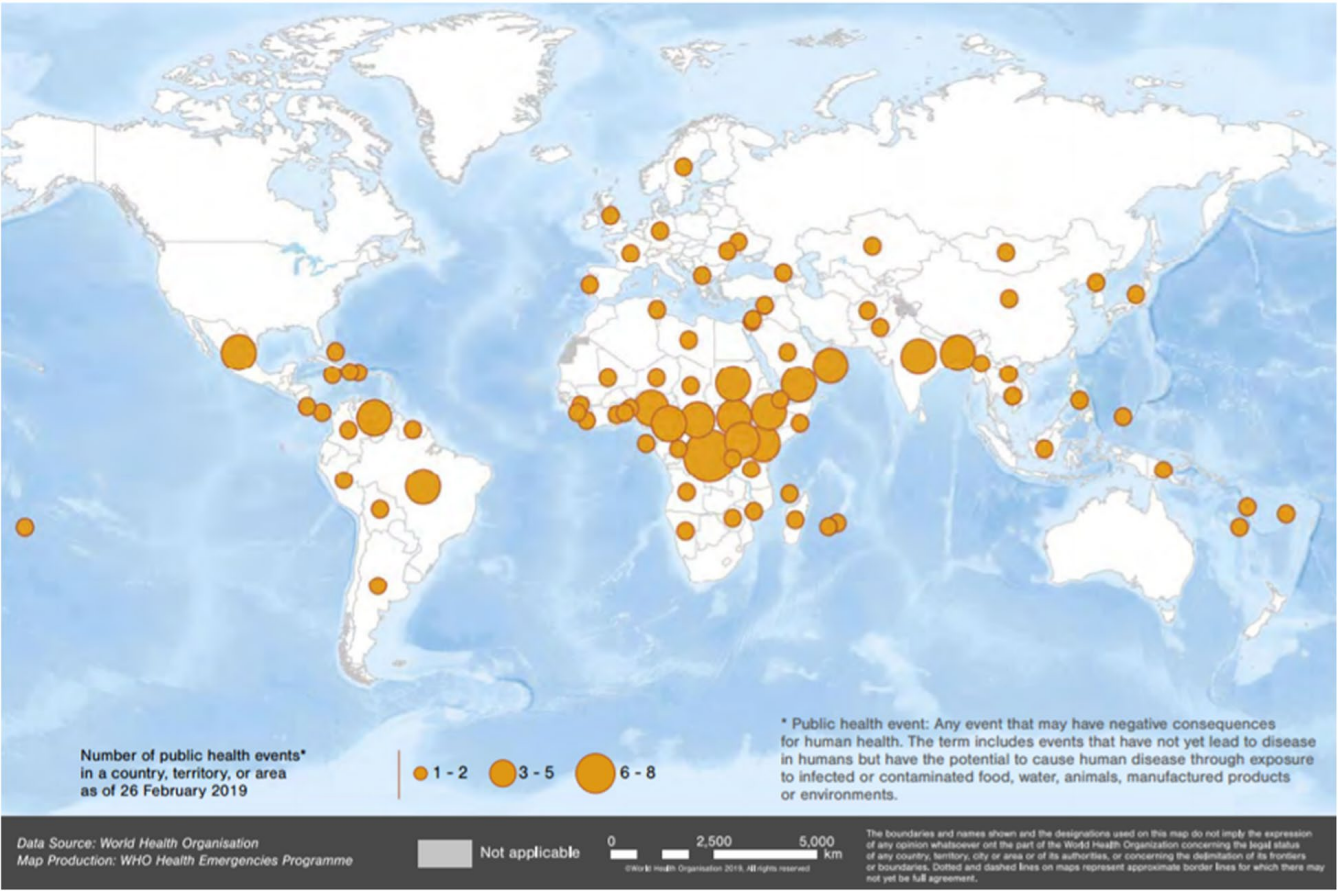
WHO’s work in emergencies: prepare, prevent, detect and respond, Annual report (2018)

According to the WHO annual report in 2018 on *Work in emergencies: prepare, prevent, detect and respond*, a total of 481 health events were reported in 141 countries and territories [78]. Some of these public health events are caused by well-known diseases but also include novel pathogens, the so-called Disease X, with great potential to cause widespread outbreaks, and often associated with high case-fatality rates, with no efficient preventive treatments or vaccines [67]. In 2019, of a total of 483 public health events detected by WHO, 22% were registered in the WHO African region (AFRO) [80]. In 2019, AFRO accounted for the highest-burden of disease outbreaks, with the most frequently reported being: cholera (20.8%), measles (11.5%), and yellow fever (7.3%). The countries that reported at least 4 health events of infectious disease outbreaks in 2018 were Uganda, Central African Republic, South Sudan, the Democratic Republic of the Congo, Zimbabwe, Niger, Namibia, Libya, Kenya, Republic of the Congo and Angola [28] (Fig. 2).

### Online learning, MOOCs and emergencies

Online learning approaches are widely used in modern pedagogy, and qualifies as a potentially effective education method across different disciplines and fields, including emergency response context ([1, 10, 21, 31, 65]). Technology development has leveraged opportunities to integrate the best online activities into instruction and bring timely, up-to-date, and evidence-based information to remote areas. It might be particularly beneficial for low-resourced settings, and emergency contexts where movement of people is restricted or impossible; thus, digital tools might serve as the main or even the only avenue to deliver life-saving knowledge. The COVID-19 pandemic forced a switch to online learning which can be seen as the biggest case of massive utilisation of digital learning in the emergency context. Some scholars even came up with a new term *“pandemic pedagogy”* to name this phenomenon [30]. In this regard, some authors argued if massive open online courses (MOOCs) were “*our saviour during COVID-19 pandemic”* [64].

Massive open online courses (MOOCs), an asynchronous type of online learning delivering content via open-access, web-based courses with recorded video lectures, online activities and interactions, have existed for more than a decade and have provided completely new ways of learning for everyone who desires to choose online means of accessing learning [20, 24, 27, 49]. They were first introduced in 2008 and have since grown into a *large* globally encompassing intervention [10]. Others argue the first true MOOC was by Sebastian Thrun and Peter Norvig in 2011 in an artificial intelligence course at Stanford,a course which drew 160,000 online registrants [40]. MOOCs have been divided into three distinct type of MOOC categories [2]:

- xMOOCs that are content-focused and expert-driven, from one-to-many models, for scalable learning.
- cMOOCs are networked, self-organized and strongly peer-to-peer formats learning.
- Hybrid MOOCs are community- and task-based social learning, that emerge in free formats and spaces from the authors.

Other types of MOOCs presented in literature, include (*adaptive-MOOC*) and adaptive hybrid MOOC (ah-MOOC).

In this paper we focus on the informal, voluntary learning dissemination in massive, online formats through MOOCs and xMOOCs, where, per Beaven et al. [2], the xMOOCs have a strong focus on the content transmission and acquisition features. Expert instruction plays a major role and the course assessment is automated. In xMOOCs, learners can take the courses fully in self-study modality without any participatory elements. The xMOOC format is not limited and could include opportunities for networking, forms such as discussion forums and joint task completion.

Mazoue [27] suggested that the xMOOC model can optimise the efficiency of knowledge acquisition as it offers a formalised approach and high-quality instructional materials that are complemented with well-defined learning objectives and assessment procedures. Learning in xMOOCs most often happens in an asynchronous learning format, which is a general term to describe forms of education, instruction, and learning that do not occur in the same place or at the same time. Thus, MOOCs have become a significant part of the teaching and learning experience not only in higher education [4, 21], but also in emergency settings, for example: refugee camps [9], COVID-19 response [6, 21, 65], and emergency nursing education (Aung *et.al*, 2020).

### Challenges of online learning and MOOCs

Digitised learning approaches, including MOOCs can offer learners advantages facilitating lifelong learning, and allowing learners to work independently at their own pace and dynamics [7]. These approaches also employ more flexibility, intuitiveness, and interactivity and can be an alternative way for adult learners as they are able to access, and revisit learning materials at any time, and from anywhere they like, and thus, do not have to choose between their professional and family lives or going back to university (Aung *et.al.*, 2020, [7, 42]). However, there are some challenges that we discuss below.

One of the biggest limitations inherent to digital technology implementation are Internet connection and digital literacy. It was shown that broadband Internet connections are often hard to access in settings like refugee camps [9, 32] or low-resource settings, where mobile phones are the most commonly used technology to access learning materials [65]. Evidence suggests that in some settings MOOCs design and format should account for poor Internet connectivity and the limitation of computer-aided learning in low-resource locations. Thus, it is recommended to develop low-bandwidth instructional elements allowing learners to access courses using their mobile phones. Moreover, Internet interruption can lead to delayed communication between student and instructor or other students and by doing so affect interactivity patterns [10, 18, 39, 64]. Finally, it is expected that both students and instructors have a certain level of digital literacy and shift their mindset from traditional learning practices to a new type of online instruction,yet it might be a challenging task [9].

According to [46], MOOCs have the potential to be powerful change agents for universities and students, but studies do not cover if these online courses are good for all, or just for most persistent, self-directed learners as their initial findings show. An empirical study in dental education that moved into online format during the pandemic suggests that this means of training could achieve equivalent or better student course performance than the same pre-pandemic in-person courses. Also, the learning retention seems to be 20–60% higher [90].

For degree-producing platforms such as Coursera, edX, and FutureLearn, numbers went to 180 million by 2020, and one third of learners who registered on a MOOC platform joined in 2020. The pandemic brought many people into online education [48]. Even though current MOOCs platforms and content may not always be accessible and inclusive for all learners, it is argued that MOOC course design should be learner-centred, with a clear course structure and engaging to learners [31, 45].

Further, offering courses in multiple languages increase enrolments to health-related learning and take off the language barrier to learning [49] that may hinder inclusive education, especially among native communities [35]. Health-related learning in languages people speak leads to a significant increase in comprehension, which in turn can help prevent, manage, and bring to end health emergencies such as outbreaks. A study done in Kenya in 2015, showed that there was a significant increase in participants’ comprehension when learning information was available in Swahili, rather than just English [51].

**Fig. 3:**
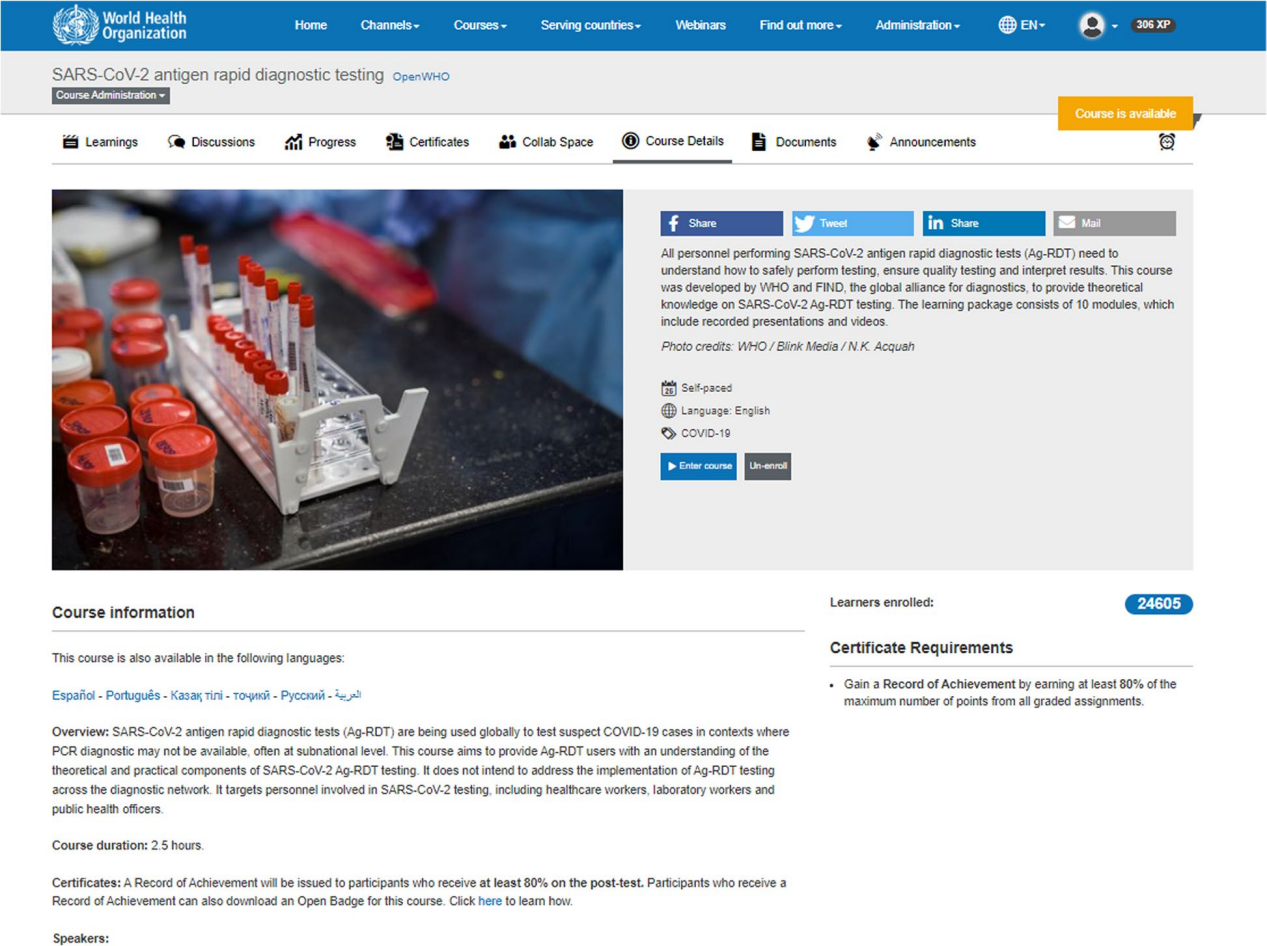
SARS-CoV-2 course landing page, screenshot

**Fig. 4:**
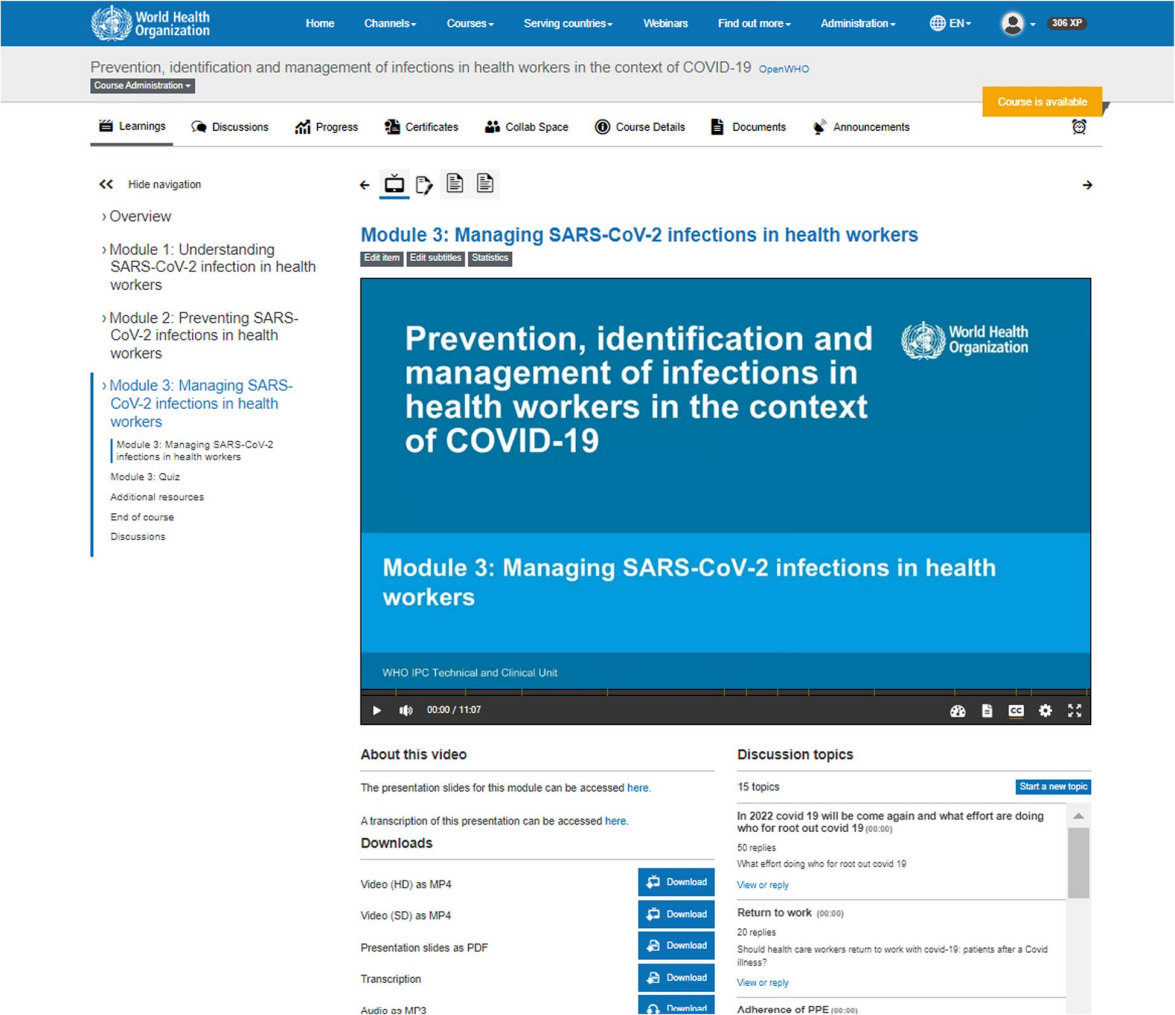
IPC Health workers course syllabus, screenshot

The courses described in this study were developed as an online asynchronous learning curriculum and they include a series of video lectures presented by technical experts, with accompanying multiple-choice questions either delivered after the video lectures or as a final graded assignment. With transcripts, slides, videos, and audio files available to download. A certificate was awarded at the end of the training to users who completed a certain threshold of the graded assignments or completed a certain percentage of the course. On average, the courses had four modules. To allow some peer-to-peer interaction, the courses’ had discussion forums, spaces where learners can interact and exchange ideas about the content and pose questions. The learning methodologies and technology applied were similar for all courses. Course materials, especially the introductory level courses, were developed considering accessibility challenges such as internet connectivity, readability and health literacy of the target population. Therefore, course content was developed and delivered for low bandwidth contexts, packaged in a way that materials could be easily downloaded while maximizing the use of visual and audio resources. Throughout the COVID-19 pandemic, findings indicated how *“just in time learning”* and presenting short informative videos were essential in providing critical knowledge during a health crisis (Figs. 3 and 4).

Since its launch, the OpenWHO team embraced the multilinguistic aspect as one of the cornerstones of their work. Even more so during the COVID-19 pandemic where the need to provide reliable information to the population was critical in the context of the emergency.

To promote the courses, the OpenWHO team relies on a variety of tools and networks – both internal and external– to make sure knowledge reaches relevant audiences. New courses and resources are frequently announced within the organization, OpenWHO learners and partners. While, externally, the OpenWHO advertises online training through a collaborative work with the WHO communication department on social media. Publications, events and webinars are also an opportunity for OpenWHO to publicise the platform and training.

## Methods

This study uses descriptive research design. Data sets were extracted from OpenWHO’s built-in reporting system for the period between April 2021 to September 2021. This system provides full course analytics such as the number of enrolments, geographical data of learners, as well as learners’ affiliation, completion, and success rates. We selected to collect only the enrolment and geographical data of all courses on infectious diseases available on OpenWHO. We then conducted an analysis by comparing the figures with the epidemiological situation and geographical distribution of the infectious diseases listed under the WHO R&D Blueprint activity. The WHO R&D Blueprint is a global strategy and preparedness plan that includes the diseases that pose the greatest public health risk due to their epidemic potential. Our analysis consisted of first, ranking countries by descending order (high to low) of the number of cases for each of the 7 infectious diseases. Next, we ranked the enrolments in infectious disease courses also by descending order. We accounted for all the courses on infectious diseases in all languages on OpenWHO. Afterwards, we examined if the highest enrolment courses (the top 5) matched the highest disease-incidence countries. This was done for the 7 infectious diseases each separately (COVID-19, Crimean Congo haemorrhagic fever, Ebola virus disease and Marburg, Lassa fever, MERS and SARS coronavirus diseases, Zika, Nipah, and Rift Valley fever). The extracted numbers allowed for identification and verification of course use in the epidemic-occurring locations and further informed on courses that are not available on OpenWHO yet and the languages of courses that should be prioritised to meet the anticipated outbreak response needs.

### Research questions

This article aims to first describe the use of OpenWHO online courses on existing priority diseases, and looks into the use of courses in countries with recent outbreaks. Secondly, the article explores the need for new learning resources for diseases with likely epidemic and pandemic potential and gives suggestions to stage languages for those courses informed by past disease burdens.

Thus, in this paper we examine the following research questions looking at them via the prism of the OpenWHO experience:

1. What is the past prevalence of the pandemic-prone diseases, where are they endemic or re-emerging and how can that inform the learning material provision?
2. How has OpenWHO learning content been used during the outbreaks?

## Results

OpenWHO work in producing online learning materials is informed by the WHO high priority R&D Blueprint disease list. Additionally, a look into languages of disease outbreaks, both past and anticipated, was given and suggestions for learning material publishing in selected languages were proposed. Within the WHO regions, the highest number of infectious disease events reported in 2018 were in the WHO African region, with Democratic Republic of the Congo (DRC), South Sudan and Uganda reporting most cases [28]. Interestingly, these three countries are the ones with the highest enrolment numbers on OpenWHO platform from the African region.

### Description of the learners´ population of the OpenWHO platform

The learners´ population analysed comprised of the participants enrolled in the OpenWHO online courses that were previously listed – all COVID-19 courses, Lassa fever, Rift valley fever, CCHF, Ebola, Zika, and MERS- making up to a total of 4.9 million enrolments. All courses on OpenWHO are accessible to the public with few exceptions not included in this study. The main target audience includes frontline healthcare professionals, health care providers, UN personnel, volunteers and students in general. OpenWHO learners’ demographics, professional affiliation, geographic distribution and languages of preference has been extensively explored in previous studies [15, 55, 58].

The OpenWHO online courses on the aforementioned infectious diseases demonstrate that complex technical information can be repurposed and simplified not only to meet the learning needs of health professionals, but also those of the general public. While measuring learning outcomes in such an environment without the use of more in-depth analytical tools is challenging, the online courses provide opportunities for learners to perform assessments, earn certificates and digital badges. The impact of these courses could be measured by analysing the high levels of engagement and the numbers of certificates awarded. Learners who completed the necessary requirements were eligible to earn a Record of Achievement certificate (to be eligible, a course participant must score a certain percentage of the points available on all course assessments, usually 80–100%) or a Confirmation of Participation certificates (to be eligible, course participants must visit a certain percentage of the items within a course, usually 100%). From the courses analysed in this study, 1.67 M of Record of Achievement certificates and 1.56 M Confirmation of Participation certificates were awarded. Furthermore, there has been reported an average course completion rate of 50% on the OpenWHO platform [57] and approximately half of all OpenWHO participants are enrolled in at least 2 courses [54]. User motivations vary from professional knowledge gaining to employer requirement or for future employment opportunities [15, 22].

Ultimately, the findings are indicative that the topical courses are being utilized frequently in the regions where the diseases occur, and in the language of preference of the affected populations. While learners in these contexts are often faced with many barriers to online learning, they are still accessing and using these materials. The design strategies implemented are the key elements to strengthen and augment the learning outcomes of the targeted audience. These strategies include providing self-paced courses, free of charge, providing materials in multiple formats and languages to address the needs and preferences of the audience.

This chapter describes high priority diseases for which there are already online training materials available on OpenWHO, revisits the needs for remaining diseases and describes the readiness and systems in place to provide a learning response to disease X. The below courses are presented in the order they were first launched on OpenWHO.

### Middle East Respiratory Syndrome (MERS) and Severe Acute Respiratory Syndrome (SARS)

From June 2012 to June 2021, a total of 2574 laboratory-confirmed cases of MERS and 886 deaths were reported, of which Saudi Arabia reported most, 2174 cases and 808 deaths (with a case-fatality ratio (CFR) of 37.2%) [84]. The largest recorded outbreak outside the Middle East was an imported case to South Korea in 2015: 185 cases were reported of which 38 resulted in death [69]. Saudi Arabia had second highest enrolments in the OpenWHO courses on MERS (*Middle East Respiratory Syndrome: Introduction* and *MERS: methods for detection, prevention, response and control*) across all language versions (English, French and Arabic), representing approximately 8.21% of the overall course enrolments, only surpassed by India, which accounts for the highest number of users in the platform overall. MERS courses are in active use based on enrolments in Jordan, United Arab Emirates, and South Korea.

OpenWHO does not have materials for SARS, which emerged in Asia in February 2003, as the platform did not exist at that time and courses have been staged for ongoing epidemic events [84]. However, respiratory syndrome disease courses have been hosted on the platform since 2019, which was rapidly repurposed into the novel coronavirus pathogen initial containment course in January 2020 as the first signs of the COVID-19 outbreak were raised. Currently, the course hosts 983,503 users across 44 languages.

### Rift Valley Fever (RVF)

Rift Valley fever, a viral zoonosis that can cause severe disease in both livestock and humans, has caused extensive outbreaks in several countries in Africa and the Arabian Peninsula: Niger, Mauritania, South Africa, Madagascar, Sudan, Kenya, Somalia, Tanzania, Egypt, Saudi Arabia and Yemen. This disease poses possible severe economic losses due to infection in livestock and the absence of an effective vaccine makes this a pathogen of considerable threat [75]. Following severe cases of disease occurring in the Africa continent, OpenWHO offered an Introductory course on Rift Valley Fever in English and French in 2017. For this course, Saudi Arabia was the second country by enrolments (4.51%). This introductory RVF course had larger numbers of users from Egypt (9th), Kenya (12th), Sudan (13th), South Africa (18th) and Yemen (30th), countries marked by their position in the list of enrolments.

### Zika virus disease

WHO declared Zika virus a Public Health Emergency of International Concern (PHEIC) on the 1st of February 2016. The outbreak has affected several countries in the Americas since 2013 [70]. Several Zika virus disease cases have been reported from Brazil since early 2015, being the country reporting the highest number of cases in the world [36]. It has been proposed that the virus might have been introduced in 2014 during the World Cup [89]. The virus was proved to be associated with birth defects such as microcephaly- Guillain–Barré syndrome [6].

The courses on Zika were offered only in English on the OpenWHO platform in 2018, when several countries reported cases. The *Zika: Introduction* course and *Risk communication for Zika virus disease* account for a total of 25, 332 enrolments. The use numbers on OpenWHO Zika courses in English are: Brazil (273 904 Zika virus disease cases—112 users on both courses), Venezuela (61,691 Zika virus disease cases, 5 users on both courses), Colombia (91,711 Zika virus disease cases, 92 users on both courses), Martinique (37,665 Zika virus disease cases), Honduras (31,468 Zika virus disease cases) as per PLISA Health Information Platform (PLISA Health Information Platform, 2020). Mexico reported 5,667 cases in 2015–2017 in pregnant women [36] and is the 4^th^ country by enrolments in the *Zika: Introduction* course (3.26%). These two courses on Zika are hosted on other platforms such as in Spanish and Portuguese in PAHO Virtual Campus and in Portuguese in two other Brazilian Health Institutes, and these totalled 162 203 enrolments as of May 2021.

### Lassa fever

Lassa fever is a viral haemorrhagic fever endemic in West Africa, affecting hundreds of thousands and provoking the death of 5,000–10,000 people every year [12, 29]. Outbreaks have occurred repeatedly in Nigeria, Liberia and Sierra Leone, as well as in Guinea and Benin [72]. In 2020, Nigeria experienced its largest recorded outbreak,a total of 6799 suspected cases, 1189 confirmed cases and 244 deaths were reported with a case fatality rate of 20.5% [34].

The online training resource *Lassa fever: Introduction*, was offered in English and French on the platform in 2018. In the English version, Nigeria users made the highest number of enrolments in the course (27.3%). For the French version, Guinea ranked second (6.9%) and Nigeria fifth (3.6%) in 2018.

### Ebola Virus Disease (EVD)

The Democratic Republic of Congo (DRC) has experienced 12 Ebola virus disease outbreaks since the disease was discovered in 1976. Several other countries have also registered Ebola outbreaks. The countries include Sierra Leone; Liberia; Guinea; Mali; Nigeria; Uganda; Sudan; Congo; Gabon [80].

French is the official language of DRC (31,900,000 speakers). There are also four national languages: Swahili (11,100,000 speakers), Luba-kasai (7,000,000 speakers), Kituba (5,000,000 speakers) and Lingala (2,040,000 speakers) [52].

At the launch of OpenWHO in 2017, one of the first courses offered was *Ebola: Knowledge resources for responders* in English and French to support the response of the 2017 Ebola outbreak in the Likati province of the DRC that was the 8th EVD outbreak in DRC [73]. The course *Ebola: Introduction* was also translated into Lingala, the language spoken in the Likati province, and is offered in English, French and Swahili to support the eastern DRC outbreaks numbers 10, 11 and 12.

**Fig. 5:**
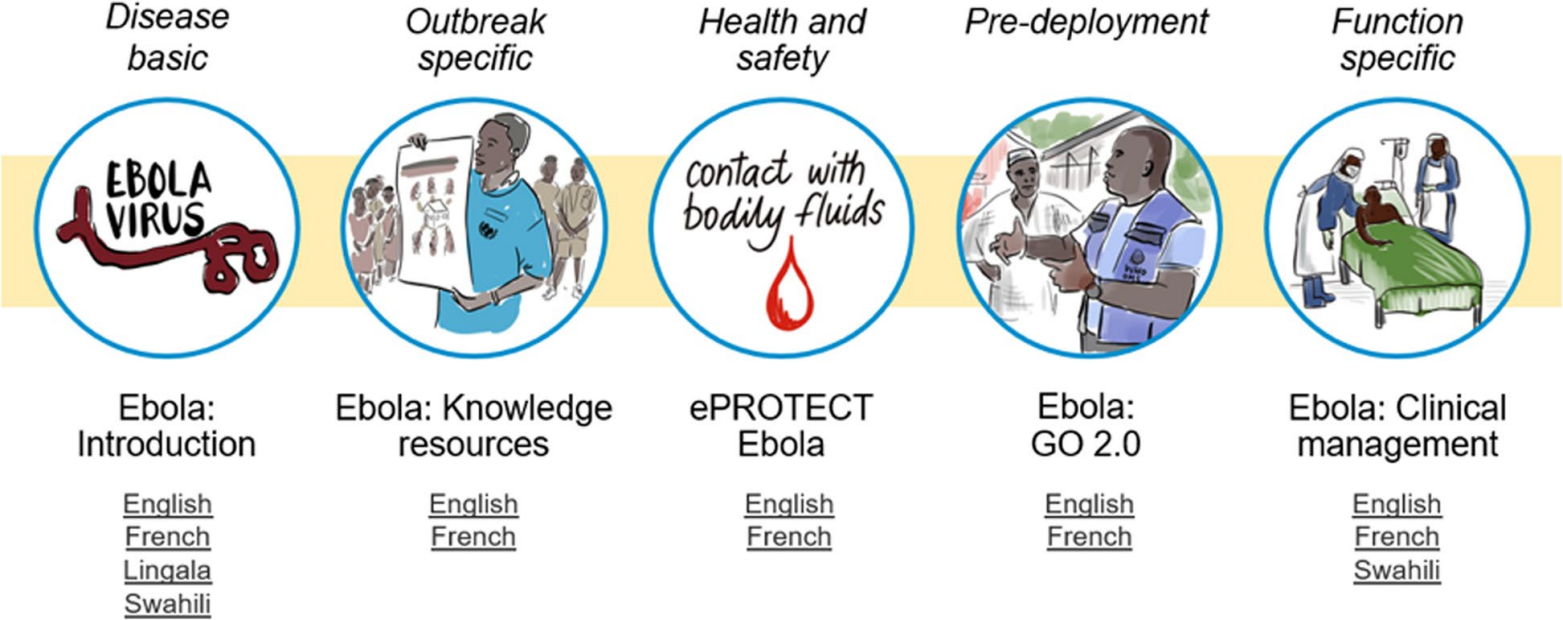
Ebola-specific courses on OpenWHO and languages versions available

All OpenWHO courses (Fig. 5) contribute to the knowledge base for the responders.

**Fig. 6:**
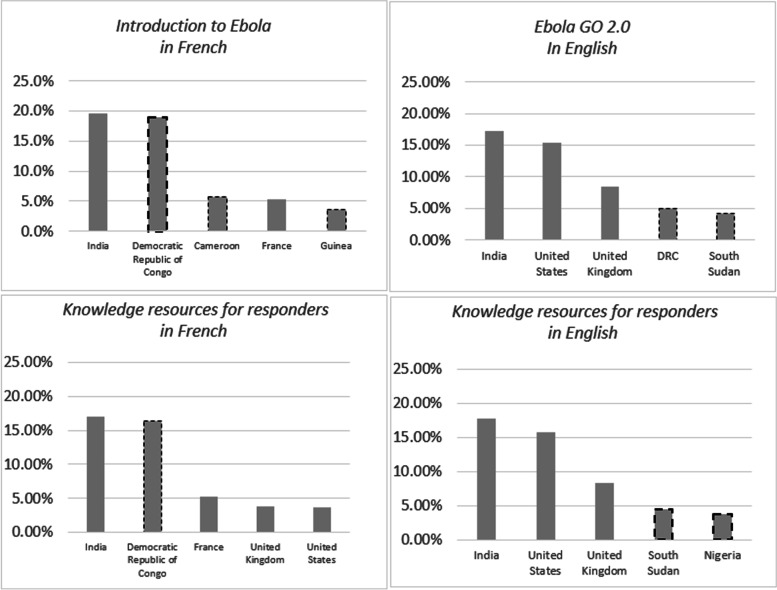
Top countries of selected OpenWHO Ebola courses

OpenWHO data shows (Fig. 6) that DRC ranks as one of the top countries among all enrolments in all Ebola-related courses in the French version: *Ebola: Introduction* (19%), *Clinical management of Ebola virus disease* (15.8%), *eProtect* (20.6%), *Ebola GO 2.0* (22.5%), and *Ebola: Knowledge resources for responders* (16.5%).

There is a high number of enrolments in Ebola courses by users from countries that share borders with DRC or other countries that have previously reported Ebola cases. For example, Cameroon (5%) and Guinea (3.6%) are listed with a high percentage of enrolments in the French versions of the *Ebola: Introduction* course. Nigeria ranks among the top countries listed in the English version of the *Clinical management of Ebola virus disease* (3.3%) and the *Ebola: Knowledge resources for responders* (3.8%). South Sudan, another DRC neighbouring country, figures among the top countries with the highest percentage of enrolments in the English version of the courses: *eProtect* (7.3%), *Ebola GO 2.0* (4.24%), and the *Ebola: Knowledge resources for responders* (4.5%).

### Crimean-Congo Haemorrhagic Fever

Crimean-Congo Haemorrhagic Fever (CCHF) is a tick-borne viral disease with no definitive treatment with a high fatality rate (10–40%) and is endemic in many countries in Africa, Asia, the Balkans, and the Middle East [68]. Most of the cases have been reported in Turkey, Russia, Iran and Uzbekistan [73]. Sporadic cases have been occurring in other countries with increased concerns regarding the recent surge of cases in Afghanistan and Pakistan. In 2017, Afghanistan reported 242 cases and 42 deaths due to CCHF (CFR 17.35%) [75]. Pakistan reported 63 cases in 2018 and 75 in 2019 [33]. Interestingly, users in Pakistan comprise 7.43% of the total enrolments in the CCHF course, placing it as the 3^rd^ country by all course enrolments.

In the online course dedicated to CCFH, there are a total of 4340 enrolments, with highest burden countries placed in the enrolment list followingly: Pakistan (7.42%); Turkey (1.13%); Iran (1.13%); Russian (0.17%); Uzbekistan (0.17%), and Afghanistan (0.74%).

### COVID-19

COVID-19 was declared a public health emergency of international concern (PHEIC) on January 30, 2020. Four days before that, OpenWHO platform launched its first COVID-19 topical course that was first named *Emerging respiratory viruses: methods for detection, prevention, response and control. Later, the course title was* changed to *Introduction to COVID-19: methods for detection, prevention, response and control*.

Massive efforts were made to expedite the production of a variety of COVID-19 courses on OpenWHO to serve the rising need of information globally. Currently, 46 different COVID-19 topics are hosted on OpenWHO, with 6 more COVID-19 topics in active production, 7 additional Solidarity Trial Vaccine courses are visible for targeted countries’ audiences only, and 5 courses have been produced by different WHO country offices. The introductory course on COVID-19, which totalled 981, 962 users across both platforms, OpenWHO and PAHO Campus virtual, has been translated into 44 languages, with more languages under production. OpenWHO reached 65 different languages by the October 2022.

**Fig. 7:**
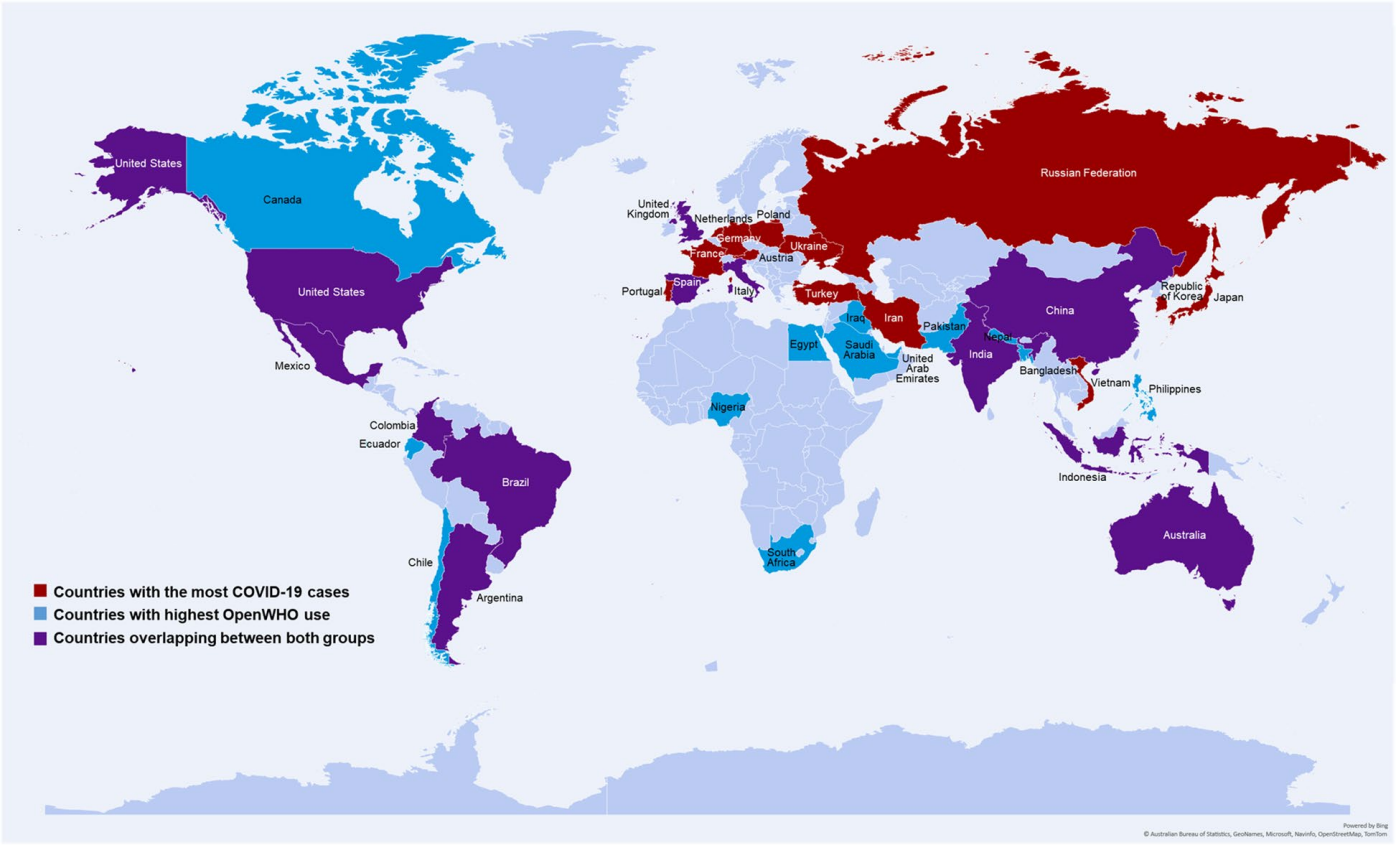
COVID-19 courses use in countries with the highest case count

The COVID-19 learning use case has followed the burden of disease during the pandemic [59] When overlaying the data of countries with the highest case count per capita and with the OpenWHO use data (Fig. 7) there are 13 countries out of 30 where the use case meets the largest burden of disease.

In a previous analysis, it was shown that the platform use accompanied some trends throughout the pandemic. It was demonstrated that the use of the platform was highest in the regions where the number of cases were the highest when the study was undertaken in August 2020. During that time, of the 20 countries with the highest number of COVID-19 cases (i.e., 82.55% of the global caseload), 14 of those countries were also among the 20 countries with the highest OpenWHO activity levels. Moreover, the findings demonstrated that users from the most affected countries accessed the Introductory course in the official language of their country [60].

### Marburg

The Marburg virus course went live in English on OpenWHO in November 2021 and in French in October 2022. The courses have 6880 learners from 167 countries. Marburg virus has a fatality rate that can go up to 88%, and outbreaks have occurred as follows:

- Angola (2005, 374 cases, 329 deaths, CFR 88%)
- DRC (1998–2000, 154 cases, 128 deaths, CFR 83%), and
- Uganda (2014, 1 case, 1 death, CFR 100%; 2012, 15 cases, 4 deaths, CFR 27%; 2007, 4 cases, 2 deaths, CFR 50%).
- Guinea (2011, 1 case, 1 death, 100% CFR)
- Ghana (2022, 2 cases, 2 deaths, CFR of 100%)

The course has use case in all affected countries and as well as in 146 other countries. For Ghana, there are 50 users registered (12%) of registrations from the WHO AFRO region. A well informed and engaged population is key as one of the measures to contain the spreading of disease [75]. Given the geographic distribution of reported cases of Marburg disease, the OpenWHO in collaboration with the WHO science lead has launched an online course on the platform with modest learner numbers from all affected countries and is assessing the language needs of countries reporting the most cases (Table 1).

**Table 1:** Three most spoken languages in the countries that reported most cases of Marburg diseases [11]

Country	Uganda		Angola		DRC	
**Top 3 most spoken languages**	Swahili	34,113,00	Portuguese	18,100,000	French	31,900,000
	Ganda	5,560,000	Umbundu	6,980,000	Swahili	11,100,000
	English	3,604,100	Kikongo	3,220,000	Luba-kasai	7,000,000

### Nipah virus

Nipah virus disease course was launched on OpenWHO in October 2022. Nipah virus as caused several outbreaks in both animals and humans in several countries, mostly in Southeast Asia in the 2000s: Bangladesh, India, Malaysia, Singapore, and Philippines. The virus has been found in fruit bats species from Cambodia and Thailand to Madagascar [75]. Case fatality rate can vary, but it can go as high as 75% [78].

In the OpenWHO Nipah course, there is use from all countries affected by the virus (India, 97 users; Philippines, 48; Bangladesh,12; Malaysia, 7, Singapore, 1). OpenWHO has considered the language needs for the current top countries where recurrent outbreaks have occurred in Table 2.

**Table 2:** Three most spoken languages in the countries that reported most cases of Nipah virus [11]

Country	Malaysia		Bangladesh		India	
**Top 3 most spoken languages**	Malay	13,500,000	Bengali	130,000,000	Hindi	633,000,000
	English	9,633,900	Chittagonian	13,000,000	English	238,260,000
	Min Nan	2,660,000	Rangpuri	10,325,000	Bengali	104,250,000

### Disease X

OpenWHO has inhouse capacity to produce courses in short notice and has to initiate priority interventions, such as the training of health staff within 3–10 days of any health event (WHO Emergency Response Framework (ERF) [73]. At the onset of the COVID-19 outbreak, WHO experts expedited the development of learning materials on that topic to bolster the response. The first course on COVID-19 was available on OpenWHO on January 26th, 2020, *Introduction to emerging respiratory viruses: methods for detection, prevention, response and control* followed the first International Health Regulations (IHR) Emergency Committee held on January 22–23, 2020. The production of this OpenWHO course was expedited in a few days.

WHO experts provided evidence to the OpenWHO team to publish initial information on the novel coronavirus and guidance on managing severe acute respiratory pathogens into a learning course. Since January 2020, the OpenWHO team has transferred essential knowledge for managing the COVID-19 pandemic to frontline responders, decision-makers and the public in a mass delivery mode.

OpenWHO’s quick adaptation to the fully remote modes of learning material development during the COVID-19 pandemic facilitated the production of several courses in different topics in dozens of languages in a record time. The same approach can also be applied to other possible future threats.

## Discussion

The analysis from the priority disease courses on Ebola, MERS, Lassa Fever and COVID-19 shows that there is strong evidence of their use in the countries of active transmission. Other disease courses show some use in countries with outbreaks.

Saudi Arabia, which has had the highest number of cases and deaths in the world since the MERS was identified in 2012, is the country with second highest enrolments on both MERS courses in all language versions available, only surpassed by India with more than 27% of all platform users.

Lassa fever course use in Nigeria, that reports recurrent outbreaks, has the highest number of enrolments in the English version, whereas Guinea, another country reporting cases of the disease, ranks second highest in enrolments in the French course.

DRC, which has been the country reporting the highest number of Ebola virus disease outbreaks since the disease was identified, is one of the top countries of enrolments in all five French Ebola courses. These findings already suggest that the usage of the language versions of each course varies and there is a noticeable preference of users to access the course on a specific disease in the national language of their country.

MOOC course design is recommended to be made in learner-centered formats with clear course structure to engage learners [31, 45]. In this regard, our findings suggest considering language as a well-documented barrier to learning [49]. Evidence suggests that social injustice and inequality were exacerbated during the pandemic, with underrepresented and/or disadvantaged learners more strongly impacted by adverse conditions [44]. Moreover, it was shown that enrolment rates of some MOOCs increased in the second quarter of 2020 among younger, less educated learners from developing countries compared to the pre-pandemic era [18], which means that MOOCs play a significant role in learning transfer in this group. Thus, this delivery format of MOOCs might be important to reach out to them in the next pandemic.

Current literature has not yet come to an agreement about learner demographic differences. Some authors reported demographics to be a poor predictor of learner performance and completion [3], while others demonstrated that there is an enrolment gap between men and women [8], and showed how age [16, 18] and country discrepancies [17, 19, 44] affect learner behaviour. All in all, we suggest that learner behaviour, including socio-demographic factors should not be considered linearly or solely, but rather be seen as multilayered, interrelated associations of factors, operating simultaneously across individual, community and policy levels.

Moreover, concerns were raised that the wide usage of digital technology may actually be widening the digital gap between different socio-demographic groups both nationally and even globally due to persistent social, economic, and political factors [41, 47]. Another example can be drawn around gender and maternal wall biases [62, 66]. Future studies should take into account these complex dynamics, and explore what it means for MOOCs design and delivery in emergencies.

An interesting phenomenon, also captured in some of the targeted disease courses, is the high number of enrolments of neighbouring countries from these high reporting countries. Although the disease is neither endemic, nor has a record of previous outbreaks in the neighbouring countries, there is still a significant number of enrolments in them. South Sudan, an anglophone country with no previous record of Ebola outbreaks, figures among the top countries with the highest percentage of enrolments in the English version of the courses: *eProtect* (7.3%), *Ebola GO 2.0* (4.24%) and the *Ebola: Knowledge resources for responders* (4.5%). The country shares 714 km of border with DRC and is well known for its intense population movements due to displacement and economic trading. Cameroon, sharing borders with countries which previously reported cases of Ebola disease, such as Nigeria, Gabon and the Congo, is listed as 4th, with 5.11% of enrolments in the French version of the *Ebola: Introduction* course. Thus, we argue that MOOC design should not only be centred around learning needs of the country of the current outbreak, but also consider neighbouring countries, taking into account possible migration flows and perceived risks of the local populations.

Some course use cases do not correlate with the disease occurrence. For instance, the Crimean-Congo Haemorrhagic Fever introductory course and the Zika resources on the platform do not have a significant number of enrolments from countries where these diseases are prevalent. One explanation could be that these courses do not host versions of languages that are spoken in the disease affected countries. Additionally, the English course is always the first to be launched, followed by the various language versions after some months, which could also impact the enrolment trend.

A large majority of the Zika affected countries were Spanish and Portuguese speaking nations, which could explain the low enrolment of users from Zika high burden regions in the English courses. We have also witnessed and recorded OpenWHO course use outside the platform itself [54]. Learning materials for some of the diseases that are not yet available on the platform, such as Nipah and Marburg, are also in active development, informed by the need to make other disease-learning materials available in the languages of the most affected populations, similar to existing courses such as Zika or Crimean-Congo haemorrhagic fever.

Other infectious disease-courses available on the platform such as cholera, monkeypox, plague, or poliomyelitis, follow the same pattern of higher enrolments from countries where these are endemic or more prevalent, however these are not covered in this article and merit dedicated research findings. In anticipation of Disease X, the OpenWHO team has prepared to have existing learning solutions and production capacity in place for any sudden unknown pathogen and situation. The way the materials were expedited at the onset of the COVID-19 pandemic, set the tone for coming threats. The anticipated learning contents include:

- Description of the causative organism of Disease X
- Description of the epidemiology of Disease X
- Explanation how Disease X is transmitted
- Description of the clinical signs and symptoms of Disease X
- Explanation how Disease X is diagnosed and treated
- Description of the key public health interventions to a Disease X outbreak

**Fig. 8:**
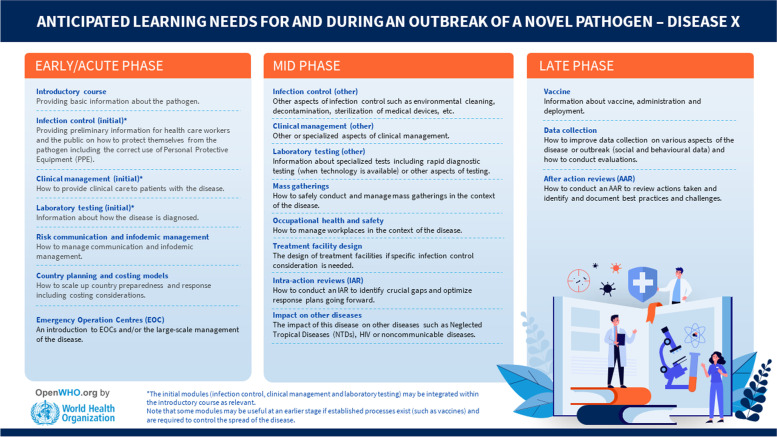
Anticipated learning contents needed for and during an outbreak of a novel pathogen – a Disease X

When anticipating future epidemic and pandemic learning delivery, a general overview of topical content of the learning is proposed (see Fig. 8).

WHO departments produced learning related to the technical guidance and OpenWHO used the power of crowdsourcing to provide the learning materials in the languages of the affected communities. The overall platform features allowing for low-bandwidth learning formats and open access, serves the needs of the affected populations. Improvements and innovations have leveraged remote learning. However, challenges such as a lack of internet connectivity, still hamper the learning process for many [24]. The online experience on OpenWHO allows the users to make use of self-paced courses at their own time and place, through downloadable audio, video and other relevant materials that can be used offline, followed by transcripts of the course presentations. The publishing formats allow course producers to work remotely in an expedited manner to contribute to the course contents in fast and remote formats.

Dedicated channels on the platform that support the country’s response to the ongoing COVID-19 outbreaks and other health threats in the country’s national languages, are already available on OpenWHO. They can be repurposed and customised to provide disease outbreaks specific learning materials according to the country’s needs. It is imperative that the health workforce, policy makers and the public are equipped with information and knowledge necessary to handle any emerging threats. For this reason, the learning materials dissemination formats must be updated and revisited to meet the needs of the learners.

## Conclusions

OpenWHO learning platform provides life-saving knowledge and resources to frontline responders working in outbreaks and health emergencies, and equally to anybody interested in learning of the diseases with epidemic and pandemic potential. The COVID-19 pandemic has shown that disease outbreaks can cause unexpected consequences. These findings inform the need and use of the learning materials in disease outbreaks. Further, the user data discussed in this article confirms a need to add offerings in the languages spoken in outbreak impacted areas.

The OpenWHO platform aims to improve the quality and delivery of its offerings, applying technologies, learner-centric approaches, and the best practices in instructional design, ensuring to keep information up to date, especially with regards to infectious diseases.

Knowledge on diseases, their containment and protection measures is critical for a well prepared and skilled health workforce, including policy makers and the public. The platform team has prioritised the production of learning for infectious diseases that are included in the R&D Blueprint priority disease list and continues to evolve the learning per the list changes and establish more formats, topics and languages for the known and unknown disease pathogens.

Asynchronous, self-paced learning intervention that is not bound to time or place is a feasible option to reach the mass audiences at any time, before the events, during and after them. Finally, findings of this study also suggest considering the utility of MOOCs in sudden-onset emergencies that affect the whole world, such as the COVID-19 pandemic. Policymakers and organisations in public outreach functions should acknowledge the need for equity in access to evidence-based and timely delivered learning materials. As such, equitable learning in health must be a global public good.

## Data Availability

All original data and metadata leading to the paper results are available.
